# Skin Endometriosis at the Caesarean Section Scar: A Case Report and Review of the Literature

**DOI:** 10.7759/cureus.2063

**Published:** 2018-01-13

**Authors:** Fatimah Alnafisah, Shaimaa K Dawa, Sherif Alalfy

**Affiliations:** 1 Obstetrics & Gynecology Department, King Saud Hospital, Saudi Arabia; 2 Histopathology, Banha University

**Keywords:** scar endometriosis

## Abstract

Cutaneous endometriosis is one of the rare gynecological conditions. Endometriosis is defined as the presence of endometrial glands and stroma outside the endometrial cavity. It commonly occurs in pelvic sites, such as the ovaries, cul-de-sac, bowel, or pelvic peritoneum. Endometriosis at the incisional scar is difficult to diagnose because of nonspecific symptoms. Usually, patients complain of pain at the site of the incision during menstruation. The main causes in most of the reported cases are obstetrical and gynecological surgeries. Endometrial tissues may be directly implanted in the scar during operation and, under hormonal stimulation, proliferate and form scar endometriosis. Diagnosis is usually made following histopathology. A wide excision is recommended to prevent recurrence. We report a case of a 33-year-old woman presenting with a brownish mass on the lateral aspect of the Pfannenstiel incision from a previous cesarean section scar. The symptoms appeared two years after her operation. The patient had cyclical pain and brownish discharge from the lesion during menstruation. Excision of the skin lesion with underlying subcutaneous tissue showed multiple, minute, firm hemorrhagic foci. Histopathology was performed and revealed a benign endometrial gland and stroma in the tissues, confirming the diagnosis of scar endometriosis. Cutaneous endometriosis is an uncommon gynecological condition and difficult to diagnose because of the nonspecific symptoms. Usually, it is confused with other dermatological and surgical diseases and delays the diagnosis and management. Surgical scar endometriosis following obstetric and gynecological procedures is more frequent recently due to an increase in the number of caesarian sections worldwide. Health care providers should suspect cutaneous endometriosis in any women with pain and a lump in the incisional scar after pelvic surgery.

## Introduction

Abdominal wall endometriosis is a rare gynecological condition and highly related to a previous history of abdominal surgery. Abdominal wall endometriosis is rare, but it is the most common site for extrapelvic endometriosis. Surgical scar endometriosis following an obstetric and gynecological procedure is more frequent recently due to an increase in the number of caesarian sections worldwide.

## Case presentation

A 33-year-old woman presented with a complaint of cyclic pain, a mass, and brownish discharge during menstruation from the lateral aspect of the Pfannenstiel incision of a previous cesarean section scar. A review of her past medical and surgical history revealed that she had undergone three lower segment cesarean sections and one laparoscopic surgery for a painful right ovarian cyst during her first pregnancy. The mass started to appear two years after her last caesarian section. The patient had cyclical pain and brownish discharge from the lesion during menstruation.

**Figure 1:**
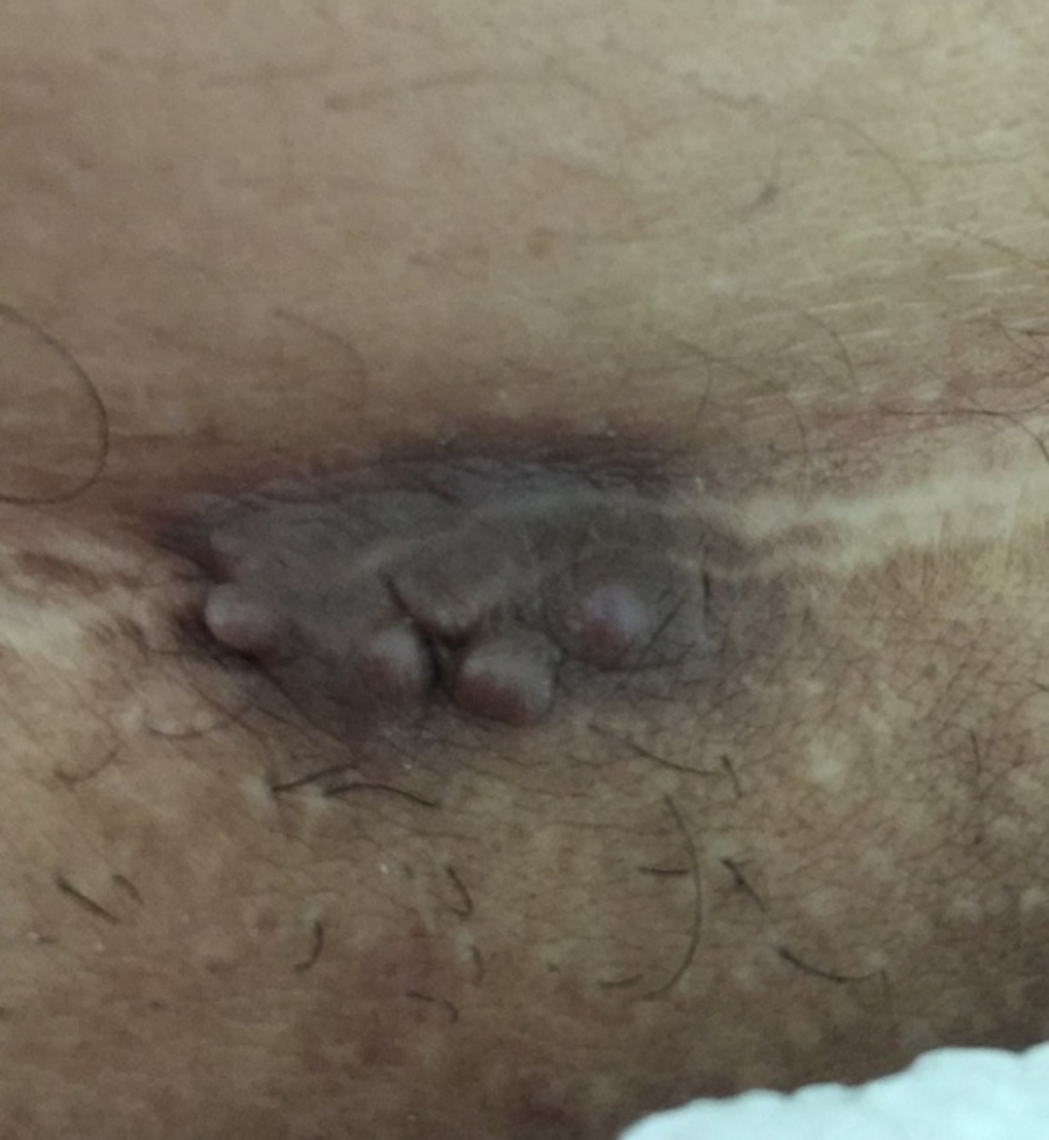
Endometrioma measuring 2 cm x 3 cm located on the lateral aspect of the Pfannenstiel incision with tiny multiple orifices protruding on the skin

A physical examination revealed a palpable, tender, subcutaneous mass, measuring 2x3 cm, located on the lateral aspect of the Pfannenstiel incision, with multiple tiny orifices protruding on the skin (Figure 1). 

**Figure 2:**
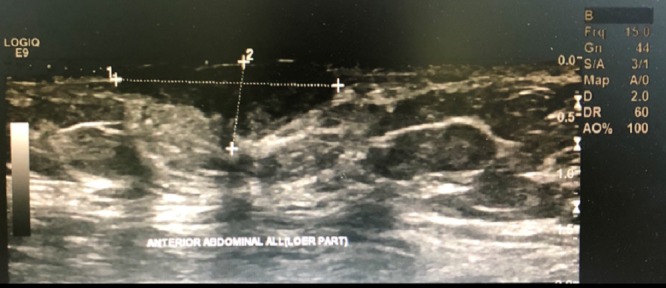
Abdominal wall soft tissue revealed a heterogeneous hypoechoic mass with few vasculatures

Sonography and Doppler examinations of the abdominal wall soft tissue revealed a heterogeneous hypoechoic mass with little vasculature (Figure 2).

**Figure 3:**
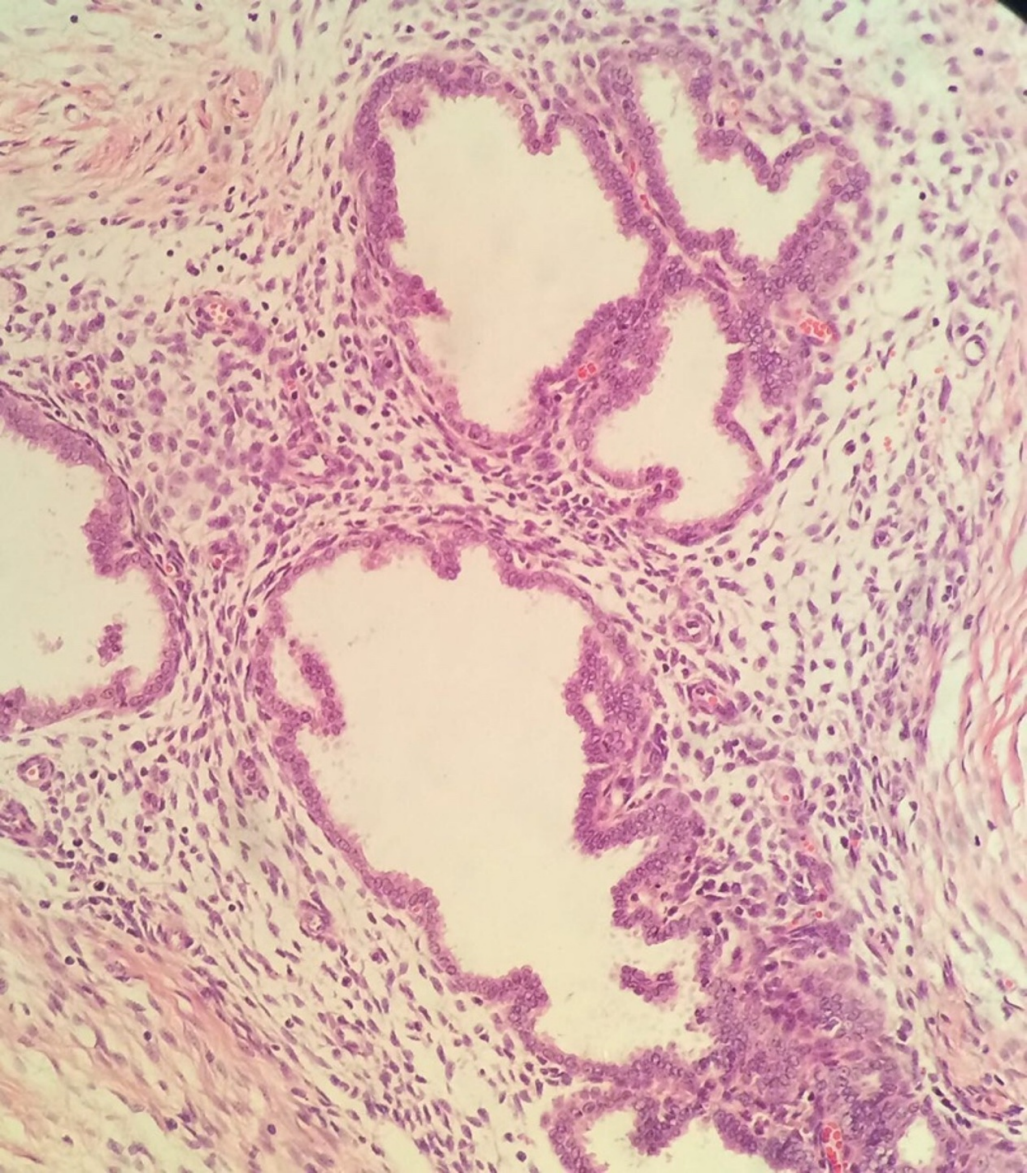
Microscopic picture of the lesion showed endometrial glands and stroma in the subcutaneous tissues of the skin

A wide local excision of the skin lesion with the underlying subcutaneous tissue, measuring 4.1x5.2 cm, found multiple, minute, firm hemorrhagic foci. Histopathology was performed and revealed a benign endometrial gland and stroma in the tissues, confirming the diagnosis of endometriosis (Figure 3). 

The postoperative period was uneventful, and periodic follow-up for 11 months yielded no recurrence.

## Discussion

Endometriosis is a chronic inflammatory reaction characterized by the presence of endometriomas outside the uterine cavity. It mainly causes painful symptoms and infertility while some women don’t experience symptoms at all. The prevalence in the general female population is 2% to 10% but reaches up to 50% in infertile women [1]. The main etiology of endometriosis is not clear, but many studies suggest the hematogenous or lymphatic spread of stem cells from bone marrow or coelomic metaplasia [2].

It commonly occurs in pelvic sites, such as the ovaries, cul-de-sac, bowel, or pelvic peritoneum. Extrapelvic endometriosis can also occur but less commonly, including sites such as the abdominal wall, lung, pleura, bladder, omentum, or bowel. It is estrogen-dependent and commonly affects women during their reproductive age.

Abdominal wall endometriosis is one of the major extrapelvic sites and usually is highly related to abdominal surgeries and obstetric and gynecological procedures [3] like a cesarean section, hysterectomy, amniocentesis, tubal ligation, appendectomies, umbilical hernioplasties, and laparoscopic trocar tracts [4]. Primary cutaneous endometriosis in the umbilicus was reported in a patient without any history of abdominal surgery [5].

Cutaneous endometriosis is known as the presence of endometrial tissues that were implanted in the skin. This is estimated to occur at approximately less than 1% of ectopic sites. It is classified as primary cutaneous endometriosis that occurs spontaneously without any previous operation and secondary cutaneous endometriosis. It most commonly occurs after abdominal surgery [5].

Most of the cases are referred to general surgeons for evaluation because they are commonly misdiagnosed as granuloma, hematoma, incisional hernia, keloid, or malignancy [4-5]. Surgical scar endometriosis following cesarean section is more frequent recently mainly due to an increase in the number of cesarean sections.

The Pfannenstiel incision of a cesarean section scar is the most common site of abdominal wall endometriosis with an incidence of approximately 0.03% to 0.4%. Some published cases suggest a high incidence after a hysterectomy due to high cellular replication from early decidua that has pluripotential effects [6].

The pathogenesis of scar endometriosis is very complicated, and the main causes in most of the reported cases are obstetric and gynecological surgeries. Endometrial tissue may be directly implanted in the scar during operation and, under hormonal stimulation, proliferate and form scar endometriosis [7]. During surgery, the uterus is opened, and cells easily move into the pelvic cavity through amniotic fluid and are transported into ectopic sites, such as the skin, subcutaneous tissues, or muscles of the abdomen and pelvis, near the scar [8]. Implanted endometrial cells at the new site are capable of proliferating due to a bloody environment and hormonal effects allowing them to grow and form a mass that leads to clinical symptoms.

Clinically the main complaint is a cyclic or non-cyclic pain, abdominal swelling around the wound, or brownish or blood-like discharge during menstruation, as in our case. In a case-control study comparing abdominal wall endometriosis to a control group, there was a significant increase in parity and body mass index with cyclic localized abdominal pain and absent dysmenorrhea in patients with a history of surgery [9].

Scar endometriosis has also been described as a painful swelling of the scar that is worse during menses. The cyclical hemorrhage that results from hormonal stimulation during the menstrual cycle is a diagnostic criterion of scar endometriosis [4]. Spontaneous endometriosis or primary cutaneous endometriosis can also lead to a brownish-colored painful mass with spontaneous bleeding during menstruation [5].

Khan et al. performed a study at the Mayo Clinic, including 2539 women with endometriosis. Of these, 34 women (1.34%) had abdominal wall endometriosis with 41% of the cases diagnosed clinically [9]. Among these cases, 59% had endometriosis at the cesarean section scar.

Diagnosis can be reached after a careful history and physical examination. Ultrasonography (US), computed tomography (CT), magnetic resonance imaging (MRI), and Doppler sonography can be used for preoperative diagnosis. The ultrasound is the first choice to evaluate abdominal and pelvic lesions. Scar endometriosis is usually described as a solid, heterogeneous hypoechoic mass, as found in our case [4]. CT and MRI can help to diagnose and exclude other lesions in the abdominal wall like a hernia, lipoma, granuloma, or tumor. Fine needle aspiration cytology (FNAC) can also be used as a preoperative diagnosis and for excluding malignancy [4]. The most accurate diagnosis is a postoperative histopathology of the specimen, showing stroma and glands in the resected tissues.

Cutaneous endometriosis is mainly treated by a wide local excision with clear margins to prevent recurrence. Medical management with hormones can relieve the clinical symptoms. The recurrence rate after surgery is generally low. In many reported studies, there was no recurrence [10].

Surgery with a wide local excision is the main method of preventing recurrence. A peritoneal wash with saline and isolation of the surgical scar may have some role in the prevention of scar endometriosis. During the closure of the superficial layer of the abdomen, change needles and replace instruments to prevent iatrogenic cellular transport to the scar [10].

## Conclusions

Cutaneous endometriosis is a rare condition and difficult to diagnose because of its nonspecific symptoms. Usually, it is confused with other dermatological and surgical diseases, delaying diagnosis and management. A few cases have been reported and these are single case reports or case series. There is no clear guideline for the diagnosis and management of such cases. Health care providers should suspect cutaneous endometriosis in any woman with pain and a lump in the incisional scar after pelvic surgery. Complete excision of the lesion is recommended. Histopathology is important to confirm the diagnosis and to exclude malignancy.
